# The Effects of Forming Parameters on Conical Ring Rolling Process

**DOI:** 10.1155/2014/235656

**Published:** 2014-08-17

**Authors:** Wen Meng, Guoqun Zhao, Yanjin Guan

**Affiliations:** Key Laboratory for Liquid-Solid Structural Evolution and Processing of Materials (Ministry of Education), Shandong University, Jinan, Shandong 250061, China

## Abstract

The plastic penetration condition and biting-in condition of a radial conical ring rolling process with a closed die structure on the top and bottom of driven roll, simplified as RCRRCDS, were established. The reasonable value range of mandrel feed rate in rolling process was deduced. A coupled thermomechanical 3D FE model of RCRRCDS process was established. The changing laws of equivalent plastic strain (PEEQ) and temperature distributions with rolling time were investigated. The effects of ring's outer radius growth rate and rolls sizes on the uniformities of PEEQ and temperature distributions, average rolling force, and average rolling moment were studied. The results indicate that the PEEQ at the inner layer and outer layer of rolled ring are larger than that at the middle layer of ring; the temperatures at the “obtuse angle zone” of ring's cross-section are higher than those at “acute angle zone”; the temperature at the central part of ring is higher than that at the middle part of ring's outer surfaces. As the ring's outer radius growth rate increases at its reasonable value ranges, the uniformities of PEEQ and temperature distributions increase. Finally, the optimal values of the ring's outer radius growth rate and rolls sizes were obtained.

## 1. Introduction

The radial conical ring rolling process with a closed die structure on the top and bottom of driven roll, simplified as RCRRCDS, is an advanced plastic forming technology. It has dominant advantages in saving material and cost and improving production efficiency. Since the die structure consisting of the driven roll and mandrel is closed in RCRRCDS process, the deformation of conical ring along the axial direction suffers from the restrictions of the top and bottom parts of driven roll. The fishtail defects usually occurring in radial-axial conical ring rolling process can be effectively avoided, and rolled rings have good dimensions and mechanical properties. Therefore, it is widely used in the fields of mechanical industry, wind power, aerospace, and so forth. The most typical conical products are nozzle supports, flanges [1], aeroengine accessory, the main part of nuclear reactor shell [2], and aluminum alloy conical ring with inner steps [3].

Recently, scholars mainly studied ring rolling processes by using simulation method, including the feeding strategies of reducing power consumption [4], reducing the maximum load [5], realizing a steady forming process [6], and the modeling of radial-axial ring rolling process [7]. Many researchers investigated the changing laws of equivalent plastic strain (PEEQ), temperature, grain size, and microstructure evolution of rolled rings in hot ring rolling or radial-axial ring rolling processes. Wang et al. [8] established a strain rate-/temperature-/microstructure-dependent constitutive model of Ti-6Al-4V alloy by the user's subroutine VUMAT of ABAQUS/Explicit software, analyzed the microstructure evolution laws in hot ring rolling of titanium alloy, and claimed that the uniformity of distribution, grain size, and volume fraction of *β* phase increase with the decrease of driven roll's rotational speed or the increase of the mandrel feed rate and the initial temperature of ring blank. Qian and Pan [9] established a coupled macro-microscopic finite element model for combining blank-forging and rolling process and obtained the evolution and distribution laws of strain, temperature, grains sizes, and dynamic recrystallization from the ingot to rolled ring. They declared that the higher temperature and larger strain are helpful for the improvement of dynamic recrystallization fraction. Zhu et al. [10] simulated a hot ring rolling process of titanium alloy by an internal state variable microstructure model for TA15 titanium alloy and pointed out that, with the increase of deformation degree or the initial rolling temperature, the primary *α* grain size distribution becomes more uniform. Zhichao et al. [11] analyzed the effects of rolling parameters of a hot ring rolling process on microstructure evolution of rolled ring based on a microstructure evolution model of AISI 5140 steel.

Zhou et al. [12] investigated the effects of rolling parameters on PEEQ and temperature distributions in radial-axial ring rolling process and declared that, with the increase of the mandrel feed rate and the initial temperature of ring blank, the PEEQ and temperature distributions of rolled ring become more uniform. Anjami and Basti [13] and Zhou et al. [14] studied the effects of rolls sizes on the PEEQ and temperature distributions, rolling force, and rolling moment in hot ring rolling and radial-axial ring rolling processes, respectively. They obtained the optimal rolls sizes for more uniform PEEQ and temperature distributions of rolled rings but did not give out the reasonable mandrel feeding strategy.

As the need for profiled rings increases in industries, the studies about profiled ring rolling processes become a hot issue. However, reports about the PEEQ and temperature distributions in profiled ring rolling processes were insufficient, especially those about conical ring rolling process. Qian et al. [16] developed a combined ring rolling process based on the minimum resistance principle of metal plastic forming in order to manufacture a thick-wall and deep-groove ring. In their study, they analyzed the metal flow laws in rolling process and obtained the PEEQ and temperature distributions laws of rolled ring by finite element method. Their simulation results were in accordance with experimental ones. Seitz et al. [1] simulated and manufactured a dish-shaped ring with a thicker ring wall and lower ring height on radial-axial ring rolling mills and pointed out that a larger height reduction of the ring can result in dishing and ring climbing. Han et al. [3] optimally designed a prorolled ring blank in the aluminum alloy hot rolling process of a conical ring with inner steps by simulation and experimental methods. Yuan [17] optimally designed a conical ring blank with inner steps of steel by simulation and experimental methods. Gong and Yang [18] simulated and analyzed the effects of mandrel feed rate on the PEEQ distribution in rolling process of a conical ring with outer steps by using the feeding strategy with a constant mandrel feed rate and pointed out that the bigger mandrel feed rate and lower driven roll's rotational speed can improve the mechanical property of rolled rings. However, all of them neither gave out the reasonable mandrel feeding strategy nor studied the effects of key forming parameters such as rolls sizes on conical ring rolling processes.

Therefore, it is significantly necessary to set up the FE model of conical ring rolling process, establish the plastic penetration condition and biting-in condition in rolling process, deduce a reasonable mandrel feeding strategy, and investigate the effects of key forming parameters on the PEEQ and temperature distributions of rolled rings, average rolling force, and average rolling moment.

This paper established the plastic penetration condition and biting-in condition in RCRRCDS process, set up a reasonable mandrel feeding strategy with a constant ring's outer radius growth rate, and deduced the reasonable value range of mandrel feed rate in rolling process. The coupled thermomechanical 3D FE model of RCRRCDS process was established based on ABAQUS software. The steady conical ring rolling process was realized by numerical simulation. The effects of ring's outer radius growth rate and the sizes of rolls on the uniformity of PEEQ and temperature distributions of rolled rings, average rolling force, and average rolling moment were analyzed.

## 2. The Mathematical Model of RCRRCDS Process

In RCRRCDS process, the radii of driven roll and mandrel, as well as the inner and outer radii of conical ring, are variable along axial direction, so at a certain time the plastic penetration condition and biting-in condition at different layer-heights are different. However, the values of feed amount per revolution at different layer-heights are the same. That is to say, an unreasonable mandrel feeding strategy (i.e., a small mandrel feed rate) may result in a certain part (i.e., the bottom part) of conical ring being forged penetrated while the other part (i.e., the top part) of conical ring not being completely forged penetrated. In this situation, ring's top radius cannot grow up successfully and the rolling process is unstable. Similarly, a bigger mandrel feed rate may make a certain part (i.e., the bottom part) of conical ring successfully be bit-in the roll gap between the driven roll and the mandrel while the other part (i.e., the top part) of conical ring hardly be bit-in. Therefore, in order to make the conical ring completely forged penetrated and simultaneously bit into the roll gap between the driven roll and the mandrel, the plastic penetration and biting-in conditions at different layer-heights must be satisfied in the whole RCRRCDS process.

### 2.1. The Plastic Penetration Condition and Biting-In Condition in RCRRCDS Process

**Figure 1:**
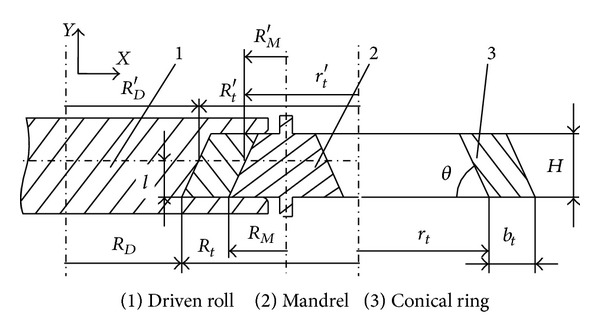
The schematic diagram of RCRRCDS process at *t* time.

Figure 1 shows the schematic diagram of RCRRCDS process at *t* time. In Figure 1, *R*_*D*_ is the bottom-radius of driven roll; *R*_*M*_ is the bottom-radius of mandrel; *R*_*t*_ is the ring's bottom outer radius at *t* time; *r*_*t*_ is the ring's bottom inner radius at *t* time; *b*_*t*_ is the thickness of ring wall at *t* time; *H* is the height of conical ring; *θ* is the included angle between ring wall and *X* axis; *R*_*D*_′ is the driven roll's radius at *l* layer-height; *R*_*M*_′ is mandrel's radius at *l* layer-height; *R*_*t*_′ is the ring's outer-radius at *l* layer-height and at *t* time; *r*_*t*_′ is the ring's inner-radius at *l* layer-height and at *t* time; *t* is the rolling time; *l* is the layer-height of conical ring. The value ranges of *l*, *t*, and *H* are (0 ≤ *l* ≤ *H*),  (0 ≤ *t* ≤ *T*_total_), and (0 < *H* ≤ *R*_*M*_tan*θ*), respectively. *T*_total_ is the total rolling time.

**Figure 2:**
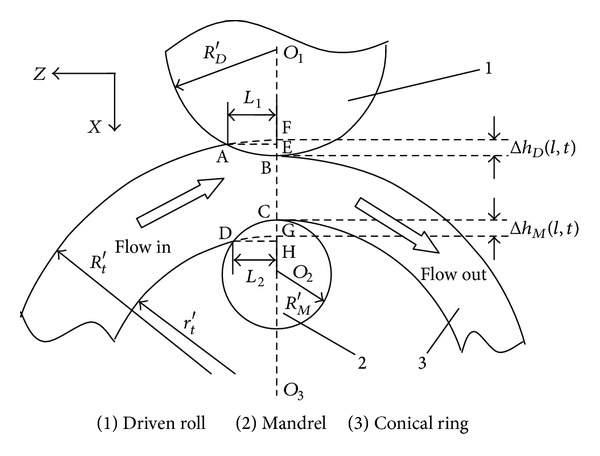
The schematic diagram of RCRRCDS process at *l* layer-height and *t* time.

Figure 2 shows the schematic diagram of RCRRCDS process at *l* layer-height and at *t* time. *O*_1_, *O*_2_, and *O*_3_ are the centers of driven roll, mandrel, and deforming ring, respectively. Δ*h*_*D*_(*l*, *t*) and Δ*h*_*M*_(*l*, *t*) are the feed amount per revolution of the driven roll and mandrel at *l* layer-height and at *t* time, respectively. *L*_1_ is the projected length of the contact circular arc *AB* between conical ring and driven roll on *z*-axis. *L*_2_ is the projected length of the contact circular arc *CD* between the conical ring and mandrel on *z*-axis.

In RCRRCDS process, since the ring suffers from the rolling forces of the driven roll and mandrel and the resistances of the top and bottom part of driven roll, the conical ring's radius continuously increases while its height is invariable. Therefore, it is reasonable to use the plastic penetration and biting-in conditions for plain ring rolling process presented by Hua et al. [19] as the beginning conditions to deduce the plastic penetration and biting-in conditions for RCRRCDS process. It must be noticed that, under the plastic penetration and biting-in conditions for plain ring rolling process, the radii of the driven roll and mandrel are both treated as constants and the inner and outer radii of ring are both treated as the function of rolling time *t*. However, under the plastic penetration and biting-in conditions at *t* time and at *l* layer-height in RCRRCDS process, the radii of driven roll and mandrel are both treated as the functions of layer-height *l*, and the inner and outer radii of ring are treated as the functions of both rolling time *t* and layer-height *l*. In this way, the plastic penetration and biting-in conditions at *t* time and at *l* layer-height in RCRRCDS process can be established as follows:

(1) Δhmin⁡l,t=6.55×10−3Rt′−rt′21RD′+1RM′+1Rt′−1rt′

(2) Δhmax⁡l,t=2β21/RD′+1/RM′21RD′+1RM′+1Rt′−1rt′

(3) Δhmin⁡l,t≤Δhl,t≤Δhmax⁡l,t,

where Δ*h*(*l*, *t*) is the feed amount per revolution at *l* layer-height and at *t* time and Δ*h*_min⁡_(*l*, *t*) and Δ*h*_max⁡_(*l*, *t*) are the permitted minimum and maximum of Δ*h*(*l*, *t*), respectively. *β* is the friction angle between the conical ring and rolls. And *R*_*D*_′ = *R*_*D*_ + *l*cot⁡*θ*, *R*_*M*_′ = *R*_*M*_ − *l*cot⁡*θ*, *R*_*t*_′ = *R*_*t*_ − *l*cot⁡*θ*, and *r*_*t*_′ = *r*_*t*_ − *l*cot⁡*θ*.

The RCRRCDS process is a problem with three-dimensional geometry and one-dimensional time. In order to make the conical ring completely forged penetrated and simultaneously bit into the roll gap between the driven roll and the mandrel, according to ((1))–((3)), the feed amount per revolution at *l* layer-height and at *t* time, Δ*h*(*l*, *t*), should satisfy the following equation:

(4) $$\begin{array}{c} {\left[\Delta h_{\min}\left(l,t\right)\right]}_{\max}\leq \Delta h\left(l,t\right)\leq {\left[\Delta h_{\max}\left(l,t\right)\right]}_{\min}, \end{array}$$

where [Δ*h*_min⁡_(*l*, *t*)]_max⁡_ is the maximum of Δ*h*_min⁡_(*l*, *t*) at *t* time and [Δ*h*_max⁡_(*l*, *t*)]_min⁡_ is the minimum of Δ*h*_max⁡_(*l*, *t*) at *t* time. By ignoring the smaller term 1/*R*_*t*_′ − 1/*r*_*t*_′ in ((2)) and ((3)), according to the monotonicity of functions Δ*h*_min⁡_(*l*, *t*) and Δ*h*_max⁡_(*l*, *t*), the maximum of function Δ*h*_min⁡_(*l*, *t*) and the minimum of function Δ*h*_max⁡_(*l*, *t*) can both be obtained at *l* = *H* as follows:

(5) Δhmin⁡l,tmax⁡=Δhmin⁡  l=H,t,Δhmax⁡l,tmin⁡=Δhmax⁡  l=H,t.

In Figure 2, according to [13] and by assuming *L*_1_ = *L*_2_ = *L*, we can get

(6) Δhl,t=L221RD′+1RM′+1Rt′−1rt′

(7) L=2Δhl,t1/RD′+1/RM′+1/Rt′−1/rt′.

In ((7)), when Δ*h*(*l*, *t*) changes with the increase of *R*_*D*_′ or *R*_*M*_′, both *L* and the contact areas between the conical ring and rolls increase. Thus, the conical ring can be more easily forged penetrated.

For convenience of statement, we represent the inner radius surface layer of rolled conical ring by using IL, the outer radius surface layer by using OL, and the middle layer by using ML, respectively. The deformation degrees at IL and OL are connected with Δ*h*_*M*_(*l*, *t*) and Δ*h*_*D*_(*l*, *t*), respectively. The bigger the Δ*h*_*M*_(*l*, *t*) is, the bigger the deformation at IL of the rolled ring is; the bigger the Δ*h*_*D*_(*l*, *t*) is, the bigger the deformation at OL of the rolled ring is. According to geometrical features of RCRRCDS process at *l* layer-height and at *t* time shown in Figure 2, the following equations can be obtained:

(8) ΔhDl,t=L221RD′+1Rt′

(9) ΔhMl,t=L221RM′−1rt′.

In order to compare the deformation at IL with OL of the rolled ring at *t* time, the difference value, *φ*(*l*, *t*), between Δ*h*_*D*_(*l*, *t*) and Δ*h*_*M*_(*l*, *t*) is adopted:

(10) φl,tΔhDl,t−ΔhMl,t=L221RD′−1RM′+1Rt′+1rt′.

In ((10)), the effect of 1/*R*_*t*_′ + 1/*r*_*t*_′ in ((10)) can be neglected, because 1/*R*_*t*_′ + 1/*r*_*t*_′ is much smaller than 1/*R*_*D*_′ − 1/*R*_*M*_′ and gradually decreases with the increase of rolling time. Thus, we can get

(11) $$\begin{array}{c} \varphi \left(l,t\right)=\frac{L^{2}}{2}\left(\frac{1}{R_{D}^{\prime }}-\frac{1}{R_{M}^{\prime }}\right). \end{array}$$

In ((11)), if *φ*(*l*, *t*) > 0, it indicates that the deformation degree at OL of rolled ring is bigger than that at IL of rolled ring at *t* time and at *l* layer-height, and vice versa.

### 2.2. The Reasonable Value Range of Mandrel Feed Rate

Determining a reasonable feeding strategy is the precondition of realizing a steady rolling process. To realize a steady and feasible radial-axial ring rolling process, the feeding strategy that can achieve a constant ring's outer radius growth rate was adopted by Guo and Yang [6], Pan [20], and Kim et al. [21], respectively. However, the mandrel feeding strategy deduced by them can only be used in radial-axial rectangular ring rolling processes but cannot be directly used in conical ring rolling process. For this reason, this paper deduced the reasonable value range of mandrel feed rate by adopting the feeding strategy with a constant ring's outer radius growth rate.

In this section, by assuming that the ring's outer radius grows up at a constant rate, the instantaneous mandrel feed rate is deduced. Thus, when mandrel rolls the conical ring by the deduced feed rate, it is thought that the conical ring's outer radius grows up at a constant rate. The deviation process of the instantaneous mandrel feed rate is as follows.

In RCRRCDS process, the driven roll rotates at a constant peripheral speed *v*_*D*_. When the constant ring's outer radius grows up at a constant rate *v*_*R*_′, the ring's outer radius at *l* layer-height and at *t* time, *R*_*t*_′, can be expressed as follows:

(12) $$\begin{array}{c} R_{t}^{\prime }=R_{0}-l\cdot \text{cot}\theta +v_{R}^{\prime }t, \end{array}$$

where *R*_0_ is the initial ring's bottom outer radius.

After heating, upsetting, and punching, the ingot is produced into a conical ring blank at first. Then the conical ring blank is rolled into a needed conical ring on ring rolling mills. According to the principle of volume constancy, in RCRRCDS process the ring's outer radius at *l* layer-height and at *t* time can be also expressed as

(13) $$\begin{array}{c} R_{t}^{\prime }=\frac{b_{t}^{\prime }}{2}+\frac{b_{0}\left(R_{0}+r_{0}-H\text{cot}\theta \right)}{2b_{t}^{\prime }}+\frac{H\text{cot}\theta }{2}, \end{array}$$

where *b*_*t*_′ is the thickness of ring wall at *l* layer-height and at *t* time and *r*_0_ and *b*_0_ are the initial inner radius and thickness of conical ring, respectively.

Because the derivative of *R*_*t*_′ with respect to time *t* is *v*_*R*_′, taking the derivative of ((13)) with respect to time *t*, the following equations can be obtained:

(14) vR′dRt′dt=vt′2R0+r0−Hcot⁡θb0bt′2−1

(15) vt′2vR′R0+r0−Hcot⁡θb0/bt′2−1,

where *v*_*t*_′ is the mandrel feed rate at *l* layer-height and at *t* time.

The mandrel feed rate at *l* layer-height and at *t* time, *v*_*t*_′, can be also approximately expressed by the average mandrel feed rate when the ring just right-rotates one revolution at *t* time:

(16) $$\begin{array}{c} v_{t}^{\prime }\approx \frac{\Delta h\left(l,t\right)}{\Delta t}, \end{array}$$

where Δ*t* is the time when the ring just right-rotates one revolution at *t* time.

By neglecting the sliding motion between the rolls and ring in RCRRCDS process, at *t* time of the rolling process, the length that the ring's bottom outer radius rotates one revolution equals the length that the radius of the driven roll rotates. Therefore, we have

(17) $$\begin{array}{c} 2\pi R_{t}^{\prime }=n_{D}R_{D}^{\prime }\Delta t, \end{array}$$

where *n*_*D*_ is the angular velocity of the driven roll.

According to ((15)), ((16)), and ((17)), it can be obtained that

(18) Δhl,t4πvR′R0′+vR′tnDRD′·bt′2R0+r0−Hcot⁡θb0−bt′2.

According to ((4)), ((16)), and ((17)), it can be obtained that

(19) $$\begin{array}{c} \frac{n_{D}R_{D}^{\prime }}{2\pi R_{t}^{\prime }}{\left[\Delta h_{\min}\left(l,t\right)\right]}_{\max}\leq v_{t}^{\prime }\leq \frac{n_{D}R_{D}^{\prime }}{2\pi R_{t}^{\prime }}{\left[\Delta h_{\max}\left(l,t\right)\right]}_{\min}. \end{array}$$

According to ((14)) and ((19)), the reasonable value range of *v*_*R*_′ can be obtained:

(20) nDRDR0+r0−Hcot⁡θb0−bt′24πRt′bt′2Δhmin⁡l,tmax⁡ ≤vR′ ≤nDRDR0+r0−Hcot⁡θb0−bt′24πRt′bt′2Δhmax⁡l,tmin⁡.

To sum up, in order to realize a steady RCRRCDS process, the instantaneous mandrel feed rate *v*_*t*_′ can be obtained by ((15)), while the reasonable value ranges of *v*_*t*_′ and *v*_*R*_′ can be obtained by ((19)) and ((20)), respectively.

## 3. The FE Modeling of RCRRCDS Process

**Figure 3:**
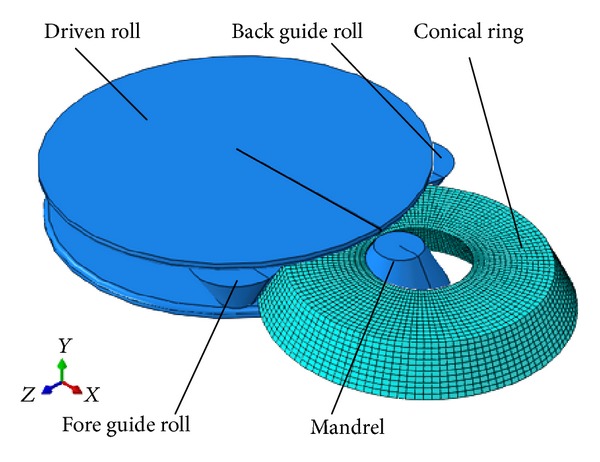
The FE model of RCRRCDS process.

According to the mandrel feeding strategy established in Section 2, the FE model of RCRRCDS process was set up based on ABAQUS/Explicit software, as shown in Figure 3. The key FE modeling techniques are expressed as follows in detail.

### 3.1. CAE Modeling

The conical ring blank is defined as a 3D deformable body; all rolls are defined as rigid bodies. The coupled thermodisplacement element with eight nodes and six faces (C3D8RT) was adopted to mesh the conical ring blank. The number of finite element meshes is approximately 8,000. Four pairs of contact relationships between the conical ring and the driven roll, mandrel, fore guide roll, and back guide roll were defined, respectively. The frictional conditions and thermal conditions such as thermal conduction, thermal convection, and thermal radiation were considered in the FE model. In order to prevent the conical ring from being blocked in the gap between the driven roll and the mandrel, the glass lubricant is usually used on the interfaces between the conical ring and the top and bottom part of driven roll. According to [22], the friction factor on these interfaces is defined as 0.1. The friction factor on the interface between the conical ring and the side face of driven roll is defined as 0.5 [23]. The friction factors on the interfaces between the conical ring with the mandrel and guide rolls are also defined as 0.5. The temperatures of driven roll and guide rolls are 100°C. The temperature of mandrel is 200°C. The initial temperature of conical blank is 900°C. The coefficient of thermal convection is 0.02 Ns^−1 ^mm^−1°^C; the thermal emissivity is 0.6 Ns^−1 ^mm^−1°^C^−4^ [24]. In order to reduce the simulation time, the mass scaling technology and dynamic explicit algorithm are employed. The mass scaling factor is selected as 50. Reduction integration and ALE remeshing technology are also adopted to avoid the nonconvergence of finite element numerical solution caused by severely distorted meshes. Hourglass control is employed to avoid zero energy modes.

### 3.2. The Motions Control of Rolls

The FE model is assembled under the global coordinate system. Each of the rolls is set to its own reference point. In the numerical simulation of RCRRCDS process, the rotational and translational motions of rolls can be controlled by setting the motion conditions of their own corresponding reference points. The driven roll rotates about its own fixed center by a constant peripheral speed. Suffering from the friction force of the conical ring, the mandrel and guide rolls can rotate about their own corresponding centers, respectively. The mandrel moves along the −*x*-axis. The *X* coordinate of mandrel at *t* time, *x*_*t*_, can be obtained according to ((15)). *x*_*t*_ = *x*_0_ − ∫*v*_*t*_′*dt*, where *x*_0_ is the *X* coordinate of mandrel at the initial time. The guide rolls move in *XOZ* plane and the movement locus (displacement-time relationship curves) of guide rolls can be obtained according to [25].

### 3.3. Material Constitutive Model

The material of the conical ring is selected as Ti-6Al-4V. Its destiny and Poisson's ratio are 4430 kg/m^3^ and 0.3, respectively. Its temperature-dependent physical properties such as thermal conductivity, specific heat, and Young's modulus are from [24]. Its constitutive model within the forging temperature range 800°C~950°C is from [26]:

(21) σ=kZmλn1−e−βελ=λ0Zq+1−λ0Zqe−αεZ=ε˙eQdefm/RT,

where *σ* is the flow stress; *Z* is the Zener-Hollomon parameter; *λ* is an internal state variable; *ε* is the plastic strain; $\dot{\varepsilon }$ is the strain rate; *Q*_defm_ is the deformation activation energy for Ti-6Al-4V; *R* is the universal gas constant; *T* is the absolute temperature; *k* is a scaling constant; *m* is the strain rate sensitivity; *n* is the structure-stress exponent; *β* is an exponential damping constant relating strain and stress; *λ*_0_ is a scaling constant related to the steady-state stress; *q* is the rate sensitivity of structure; *α* is an exponential damping constant relating strain and structure. The values of the above coefficients are shown in Table 1.

## 4. Validation of the FE Model of RCRRCDS Process

### 4.1. Theoretical Validation

Here, the energy balance rule is adopted to theoretically validate the reliability of the established FE model. Generally, if the ratio of the FE model's kinetic energy (ALLKE) to its internal energy (ALLIE) is smaller than 5%–10% in rolling process and the changing curves of ALLKE and ALLIE with rolling time are smooth, the established FE model is theoretically reliable. In RCRRCDS process studied in Section 5.1 of this paper, the bottom radii of the driven roll and mandrel are 200 mm and 100 mm, respectively. The ring's outer radius growth rate *v*_*R*_′ is 6 mm/s. Other forming parameters are shown in Table 2. According to ((20)), the reasonable value range of *v*_*R*_′ is 1.215 mm/s < *v*_*R*_′ < 6.65 mm/s. Thus, the plastic penetration and biting-in conditions in the entire RCRRCDS process can be satisfied. Figure 3 shows the established FE model of RCRRCDS process.

**Figure 4:**
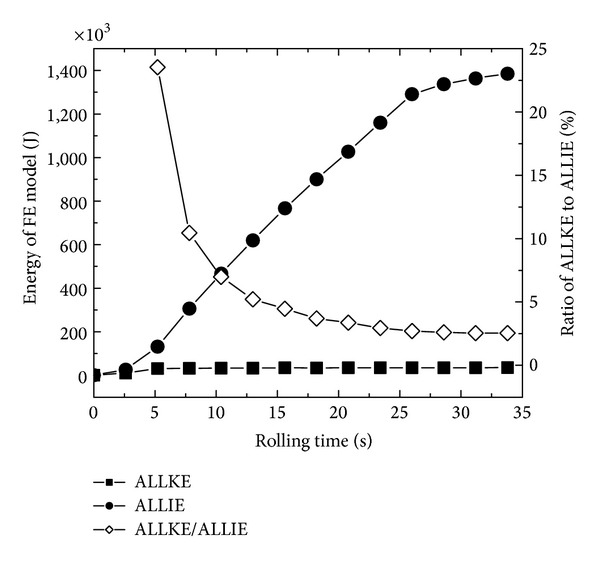
The changing curves of the kinetic and internal energies of the FE model with rolling time.

Figure 4 shows the changing curves of ALLKE and ALLIE and the ratio of the FE model's kinetic energy to its internal energy (ALLKE/ALLIE) with rolling time. It can be seen that the ratio of the FE model's kinetic energy to its internal energy is smaller than 5%–10% in the main rolling process. Therefore, the established FE model of RCRRCDS process is theoretically reliable.

### 4.2. Experimental Validation

The established RCRRCDS process is similar to the conical ring rolling experiments in [15]. In order to experimentally validate the reliability of the established RCRRCDS process, this paper established the corresponding FE model of RCRRCDS process according to the experimental situation and the parameters in [15]. The RCRRCDS experiments were carried out on D51-160A hot ring rolling mill. The experimental forming parameters are shown in Table 3. The material of conical ring blank is lead. Its density, Young's modulus, and Poisson's ratio are 11340 kg/m^3^, 16 GPa, and 0.3, respectively. Its constitutive model is *σ* = 25.06*ε* ^0.241^ [15]. In the validated model in this paper, the friction factor between conical ring blank and rolls is 0.5. The mass scaling factor is 50.  Table 4 shows the comparison of simulation and experimental results, where the numerical error between simulation results and experimental ones is defined as: (simulation value − experimental value)/experimental value.

It can be seen from Table 4 that the simulation values are close to experimental ones. The maximum and minimum numerical errors are 6.2% and 0.7%, respectively. This indicates that the FE model of RCRRCDS process established in this paper is reliable.

## 5. Results and Discussion 

This section simulated and analyzed the effects of key forming parameters on RCRRCES process based on the FE model of RCRRCDS process established in Section 3. In accordance with different research objectives, the numerical simulation experiments are made up of the following four cases.

**Case 1 .**

Investigate the changing laws of the PEEQ and temperature distributions with rolling time. Select *R*_*D*_ = 200 mm; *R*_*M*_ = 100 mm; *v*_*R*_′ = 6 mm/s, while other parameters are listed in Table 2 and keep invariable.

**Case 2 .**

Investigate the effects of ring's outer radius growth rate on the PEEQ and temperature distributions in RCRRCDS process. Select *R*_*D*_ = 200 mm; *R*_*M*_ = 100 mm; *v*_*R*_′ = {3 mm/s; 4 mm/s; 5 mm/s; 6 mm/s}, while other parameters are listed in Table 2 and keep invariable.

**Case 3 .**

Investigate the effects of driven roll radius on the PEEQ and temperature distributions in RCRRCDS process. Select *R*_*M*_ = 100 mm; *v*_*R*_′ = 4 mm/s; *R*_*D*_ = {100 mm; 200 mm; 400 mm; 600 mm; 800 mm}. In order to maintain the driven roll rotating at a constant peripheral speed, the angular velocities of the driven roll are accordingly selected as 10 rad/s, 5 rad/s, 2.5 rad/s, 1.67 rad/s, and 1.25 rad/s, respectively, while other parameters are listed in Table 2 and keep invariable.

**Case 4 .**

Investigate the effects of mandrel radius on the PEEQ and temperature distributions in RCRRCDS process. Select *R*_*D*_ = 200 mm; *v*_*R*_′ = 4 mm/s. Since *R*_*M*_ should be smaller than the initial bottom inner radius of the conical ring blank (150 mm) and bigger than 50 mm by considering that *R*_*M*_′ decreases along axial direction according to geometrical features in Figure 1, therefore, the values of *R*_*M*_ are, respectively, selected as *R*_*M*_ = {70 mm; 80 mm; 90 mm; 100 mm; 110 mm}, while other parameters are listed in Table 2 and keep invariable.

Here, the standard deviation of equivalent plastic strain (SDP) and the standard deviation of temperature (SDT) are employed to evaluate the uniformity of PEEQ and temperature distributions of rolled conical rings, respectively:

(22) SDP=∑i=1NPEEQi−PEEQa2·Vi∑i=1NViSDT=∑i=1NTi−Ta2·Vi∑i=1NVi,

where PEEQ_*a*_ is the average value of PEEQ, PEEQ_*a*_ = ∑_*i*=1_^*N*^(PEEQ_*i*_ · *V*_*i*_)/∑_*i*=1_^*N*^ *V*_*i*_; PEEQ_*i*_ is the PEEQ of element *i*; *V*_*i*_ is the volume of element *i*; *N* is the total number of elements of the conical ring; *T*_*a*_ is the average value of temperature, *T*_*a*_ = ∑_*i*=1_^*N*^(*T*_*i*_ · *V*_*i*_)/∑_*i*=1_^*N*^ *V*_*i*_; *T*_*i*_ is the temperature of element *i*. The lower the SDP is, the more uniform the PEEQ distribution is. The lower the SDT is, the more uniform the temperature distribution is.

### 5.1. The Changing Laws of PEEQ and Temperature Distributions of Rolled Ring with Rolling Time

The whole RCRRCDS process is made up of four rolling stages: initial biting-in, steady forming, final rolling, and finishing rolling round stages. The initial biting-in stage is from the time when the mandrel starts to roll the ring to the time when the ring just right-rotates the first one revolution. The steady forming stage is from the end of the initial biting-in stage to the time when the mandrel stops feed movement. The final rolling stage is from the end of the steady forming stage to the time that the mandrel goes on rolling ring another one revolution. The finishing rolling round stage is from the end of the final rolling stage to the time that the mandrel goes on rolling ring two additional revolutions. According to the simulation conditions of Case 1, the durable time of the initial biting-in stage is calculated as 3.77 s; the time of the steady forming stage is 21.83 s; the time of the final rolling stage is 2.77 s; the time of the finishing rolling round stage is 5.53 s; and the total rolling time is 33.9 s.

**Figure 5:**
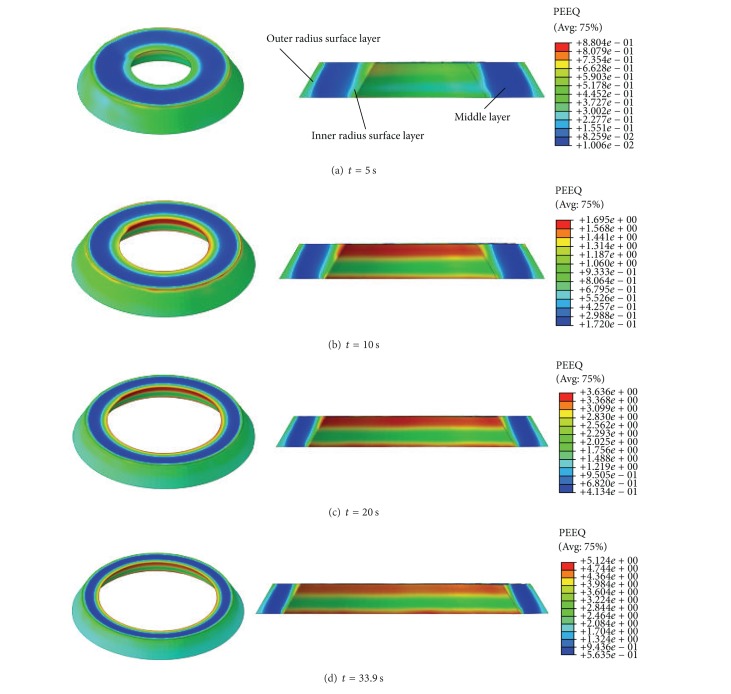
The PEEQ distributions of deforming ring at different rolling times.

Figure 5 shows the PEEQ distributions of the deforming ring at different rolling times. It can be seen that the deformation of the rolled ring mainly concentrated on the inner radius surface layer (IL) and outer radius surface layer (OL); the usually seen fishtail defects do not appear at the top and bottom part of conical ring. Generally speaking, if the initial billet geometry has sharp edges, the singularity issues such as meshes distortions may occur at the ring's sharp edges positions. However, for the RCRRCDS process studied in this paper, the singularity issues do not appear. The main reason may be that, on the one hand, the closed die structure shape of driven roll, reasonable mandrel feed rate, and the usage of reduction integration and ALE remeshing technology can effectively avoid the singularity issues; on the other hand, according to [15], the bigger the ratio of the initial conical ring blank's height to its diameter is, the more serious the distortion of rolled ring along the radial direction is, and vice versa. In this paper, the ratio of the initial conical ring blank's height to its diameter is so small (only approximately 0.144) that the distortion of the rolled ring is too tiny. Therefore, the singularity issues do not appear.

In Figure 5, with the increase of rolling time, the maximum PEEQ appears at the top and bottom part of IL. The reason is that the metal at the top and bottom part of IL suffers from the resistances of the top and bottom part of driven roll and can only be seriously deformed along the circumferential direction. These positions are the potential areas where cracks may occur due to their large and severe deformation.

**Figure 6:**
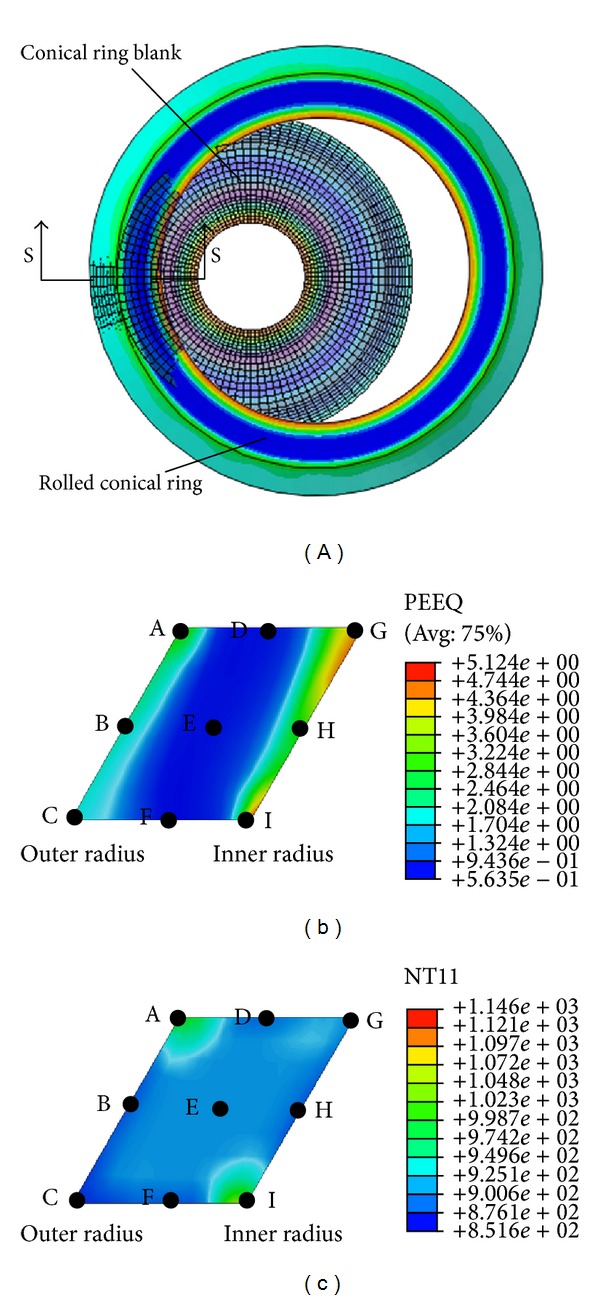
(a) Conical ring blank and rolled conical ring; (b) the PEEQ distribution at the cross-section “S-S” of rolled conical ring at *t* = 33.9 s; (c) the temperature distribution at the cross-section “S-S” of rolled conical ring at *t* = 33.9 s.

Figure 6(a) shows the conical ring blank and the rolled conical ring. Since the PEEQ and temperature distributions of the conical ring are axis-symmetric about its center, a certain cross-section of rolled conical ring “S-S” is selected to investigate the PEEQ and temperature distributions at *t* = 33.9 s, as shown in Figures 6(b) and 6(c). In Figure 6(b), the PEEQ at IL and OL are bigger than that at the middle layer (ML). The reason can be explained as follows. On the one hand, since the conical ring suffers from the rolling forces of driven roll and mandrel along radial direction and the tangential stress along the circumferential direction, there are a contracted metal flow tendency along the radial direction and expanded tendency along the axial and circumferential directions. On the other hand, since the conical ring suffers from the restrictions of the top and bottom part of the driven roll, the metal flow tendency along the axial direction is restricted. In this situation, the ring radius continuously increases, the thickness of ring wall gradually decreases, and the height of the ring is invariable. The large deformation mainly concentrates on IL and OL of the rolled ring. It also can be seen that the PEEQ at *l* layer-height of IL is larger than that of OL. The reason is that *φ*(*l*, *t*) < 0 according to the conditions of Case 1 and ((11)). This means that the feed amount of the driven roll at *l* layer-height is smaller than that of the mandrel and the larger deformation locates at IL of the rolled ring.

**Figure 7:**
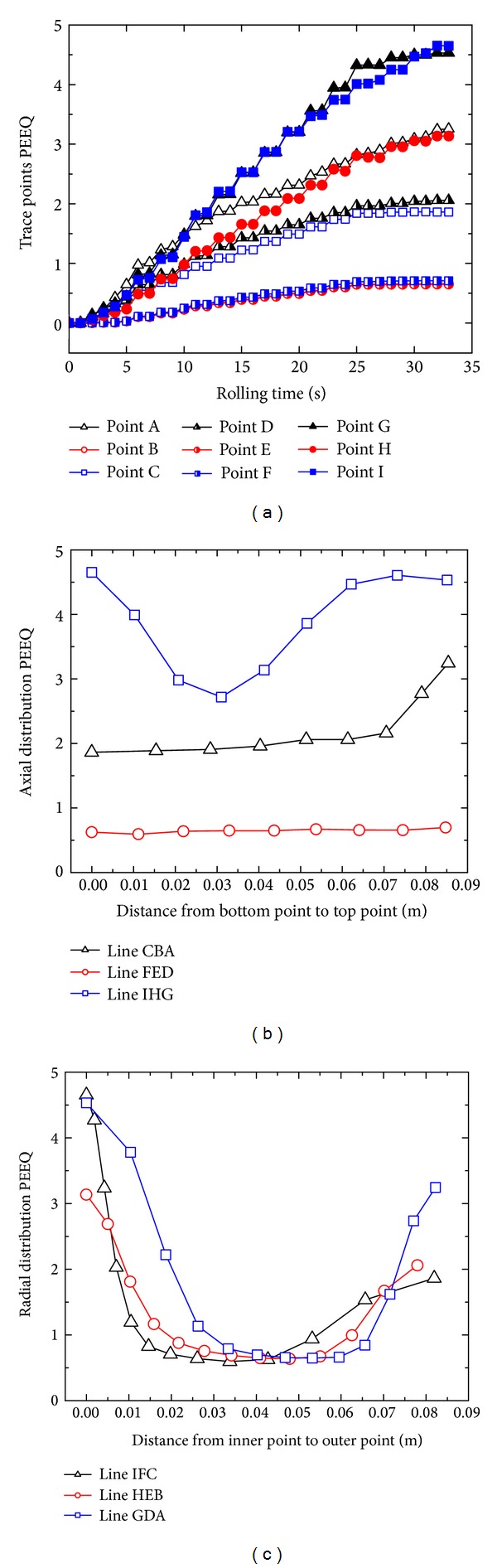
(a) The changing curves of the PEEQ of points A–I with rolling time; (b) the changing curves of PEEQ along the axial direction at *t* = 33.9 s; (c) the changing curves of PEEQ along the radial direction at *t* = 33.9 s.

Nine tracking points A–I at the cross-section “S-S” of the rolled ring are selected to investigate the PEEQ and temperature distributions with rolling time, as shown in Figures 6(b) and 6(c).  Figure 7(a) shows the changing curves of the PEEQ of points A–I with rolling time.

It can be seen from Figures 5 and 7(a) that, at the initial biting-in stage and steady forming stage, the PEEQ of points B–I basically increase linearly, while the PEEQ of point A increases fast at first and then slower. At the final rolling and finishing rolling round stage, the PEEQ of points A–I are invariable. This indicates that, with the increase of the rolling time, the uniformity of PEEQ distribution gradually decreases at the initial biting-in stage and steady forming stage and then keeps unchanged at the final rolling stage and finishing rolling round stage.

Figures 7(b) and 7(c) show the changing curves of PEEQ at the cross-section “S-S” of the rolled ring along the axial and radial directions at *t* = 33.9 s, respectively. It can be seen from Figures 6(b) and 7(b) that the PEEQ at IL (line-IHG), OL (line-CBA), and ML (line-FED) of the rolled ring reduce in turn. Along the axial direction from the ring's bottom to ring's top, the PEEQ at the ML (line-FED) of the conical ring is basically unchanged; the PEEQ at IL (line-IHG) decreases at first and then increases and finally keeps unchanged; the PEEQ at OL (line-CBA) increases slowly at first and then fast. This indicates that the larger PEEQ appears at the top and bottom of IL and the top of OL. The reason is that the metal at points A, G, and I suffers from the restriction of the top and bottom part of the driven roll and can only flow along the circumferential direction with a larger resistance, which leads to serious deformation at these positions. It also can be seen that the PEEQ at point C is smaller than those at points A, G, and I. The reason may be that the conical ring radius at point C is larger than those at points A, G, and I and thus there may exist a larger geometric space along circumferential direction for the deformed metal flow, which can reduce the PEEQ at point C to some extent.

It can be seen from Figures 6(b) and 7(c) that, along the radial direction from the inner radius to outer radius, the PEEQ of the bottom layer (BL, line-IFC), top layer (TL, line-GDA), and middle layer (ML, line-HEB) of the rolled ring decrease at first and then keep unchanged and finally increase. The PEEQ of rolled ring significantly changes along radial direction. This may be because the metal at IL and OL of conical ring is more easily forged penetrated than that at ML of conical ring. It can be deduced that the grain sizes at the positions with large PEEQ (points A, G, and I) are fine and the mechanical properties at these positions are better. However, these positions are the potential areas where cracks may occur due to their large and severe deformation.

**Figure 8:**
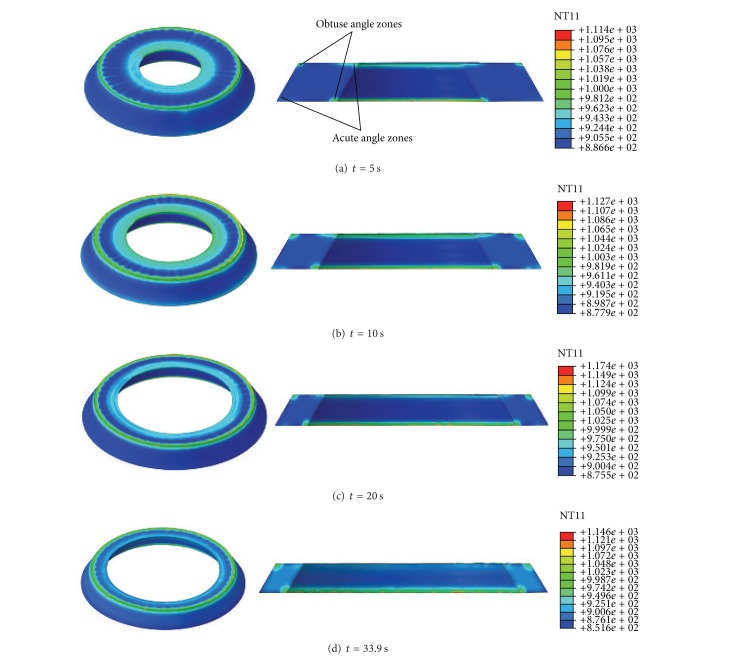
The temperature distributions of deforming ring at different rolling times.

Figure 8 shows the temperature distributions of the deforming ring at different rolling times. It can be seen from Figures 8(a)–8(d) that at the beginning of RCRRCDS process the temperatures at the top and bottom part of IL and the top part of OL are higher than those at other positions as shown in Figures 8(a) and 8(b), while at the end of RCRRCDS process the temperatures at the bottom part of IL and the top part of OL are higher than those at other positions as shown in Figures 8(c) and 8(d).

**Figure 9:**
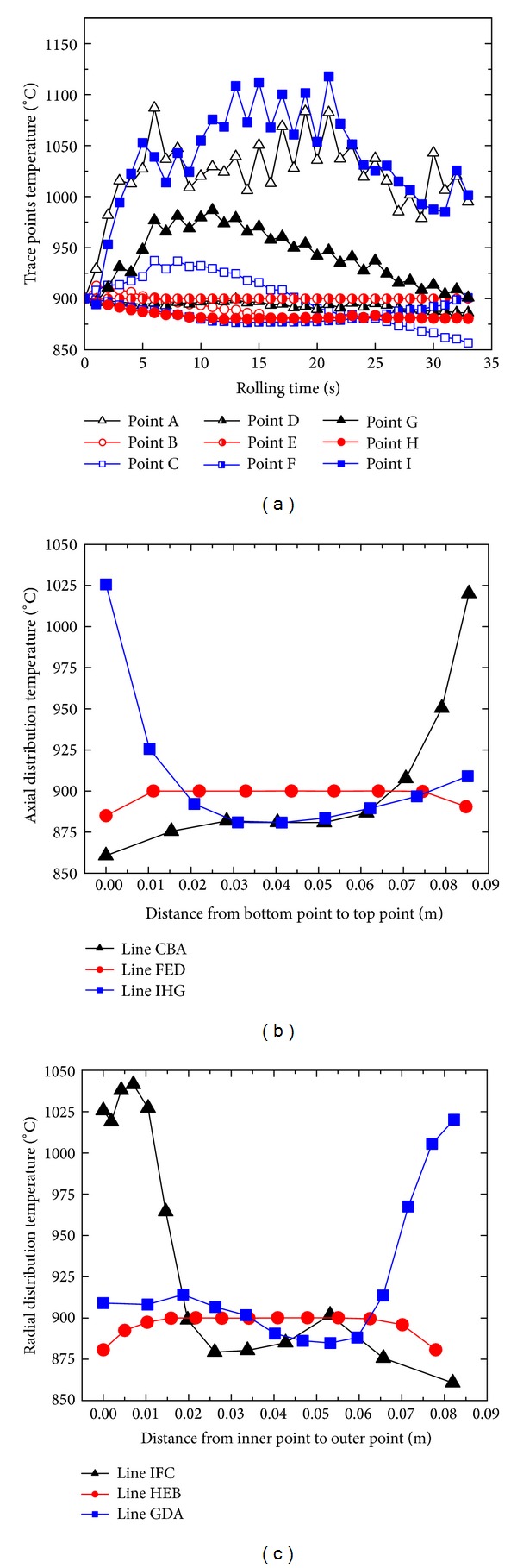
(a) The changing curves of the temperature of points A–I with rolling time; (b) the changing curves of temperatures along the axial direction at *t* = 33.9 s; (c) the changing curves of temperatures along the radial direction at *t* = 33.9 s.

Figure 9(a) shows the changing curves of the temperature of points A–I with rolling time. It can be seen from Figures 8 and 9(a) that, at the initial biting-in stage, the temperatures of points A and I rapidly increase due to the deformation heat. At the steady forming stage, the temperatures of points A and I basically keep unchanged because the deformation heat approximately balances the heat loss. At the final rolling stage and finishing rolling round stage, the temperatures of points A and I gradually decrease because there are not deformation heat while the heat loss still exists. At the initial biting-in stage, the temperatures of points C and G gradually increase due to larger deformation heat. At the steady forming stage, final rolling stage, and finishing rolling round stage, the temperatures of points C and G obviously decrease because the heat loss at these positions is larger than the deformation heat. The temperatures of points B, C, E, F, and H change a bit at the temperature range between 875°C and 900°C. It can be concluded that the uniformities of temperature distributions at the corners of conical ring's cross-section become worse, which may be caused by both uneven deformation and inhomogeneous heat loss.

Figures 9(b) and 9(c) show the changing curves of temperatures at cross-section “S-S” of rolled ring along the axial and radial directions, respectively. Figures 5(c) and 8(b) show that, along the axial direction from the ring's bottom to ring's top, the temperature at OL (line-CBA) increases slowly at first and then fast; the temperature at ML (line-FED) increases slowly at first, then keeps unchanged, and finally decreases; the temperature at IL (line-IHG) decreases sharply at first and then gradually increases.

It can be seen from Figures 6(c) and 9(c) that along radial direction from the ring's inner radius to ring's outer radius, the temperature at BL (line-IFC) decreases sharply at first, then keeps unchanged, and finally decreases again; the temperature at ML (line-HEB) slightly increases at first, then keeps unchanged, and finally decreases a little; the temperature at TL (line-GDA) decreases slowly at first and then increases sharply.

From Figures 6(c), 9(b) and 9(c), it can be seen that the temperature distribution at the central part of the cross-section of rolled ring is uniform while the temperature distributions at the edges and corners of rolled ring differ quite a lot. The temperatures at the “obtuse angle zone” (as shown as points A and I in Figure 6(c)) of the conical ring's cross-section are higher while the temperatures at the “acute angle zone” (as shown as points C and G in Figure 6(c)) of the conical ring's cross-section and at the middle part of the ring's surfaces are lower. This phenomenon can be explained by the following two aspects. On the one hand, the larger deformation concentrated on IL and OL of the conical ring results in the larger deformation heat at these positions. On the other hand, since the specific surface areas at points A and I are smaller than those at points C and G, the heat losses at points A and I are less than those at points C and G. To sum up, though the larger deformations at points A, C, I, and G occur, the temperatures at the “obtuse angle zone” (as shown as points A and I) are larger than those at the “acute angle zone” (as shown as points C and G), which may lead to the residual heat tensile stress. The metal at the “obtuse angle zone” has a relatively large damaging potential.

### 5.2. The Effects of Ring's Outer Radius Growth Rate on PEEQ and Temperature Distributions

**Figure 10:**
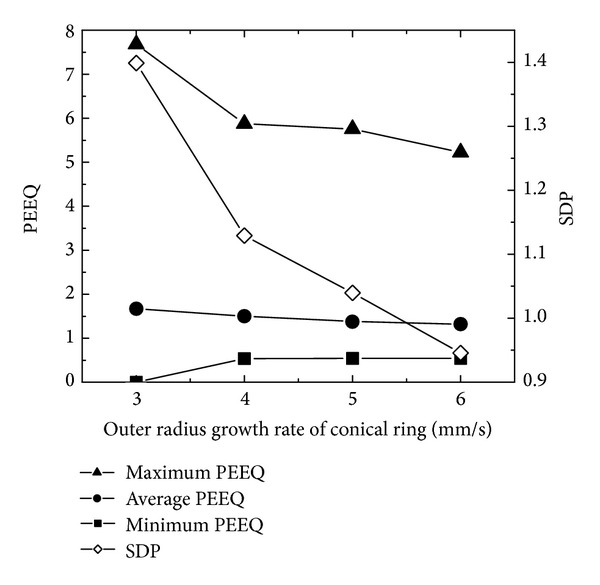
The changing curves of the maximum, minimum, and average PEEQ and SDP with ring's outer radius growth rate.

Figure 10 shows the changing curves of the maximum, minimum, and average PEEQ and SDP with ring's outer radius growth rate. In Figure 10, with the increase of the ring's outer radius growth rate, the maximum PEEQ and SDP decrease; the minimum PEEQ increases; the average PEEQ slightly decreases. The reason is analyzed as follows. Firstly, according to ((18)), when the peripheral speed of the driven roll is unchanged, the feed amount per revolution increases and the ring is more easily forged penetrated with the increase of the ring's outer radius growth rate. This phenomenon improves the uniformity of PEEQ distribution. Secondly, according to ((7)), with the increase of the feed amount per revolution, the length of the contact circular arc and the contact area between the conical ring and rolls increases. This phenomenon makes the metal more easily forged penetrated.

**Figure 11:**
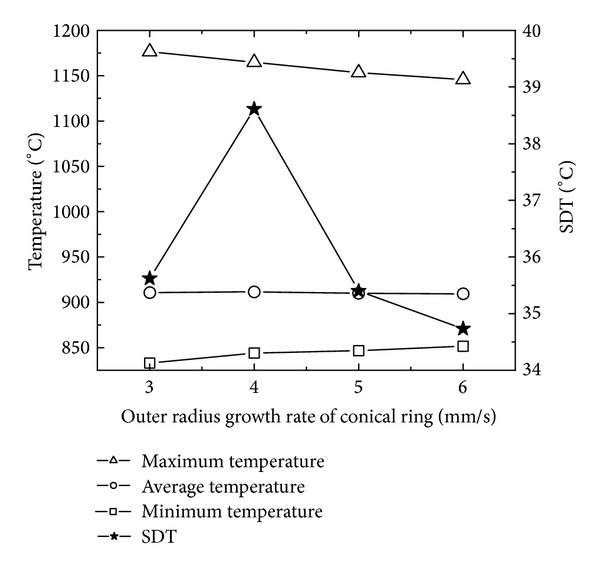
The changing curves of the maximum, minimum, and average temperatures and SDT with ring's outer radius growth rate.

Figure 11 shows the changing curves of the maximum, minimum, and average temperatures and SDT with ring's outer radius growth rate. In Figure 11, with the increase of the ring's outer radius growth rate, the minimum temperature gradually increases. This is because when the total feed amount of the mandrel is unchanged, with the increase of the ring's outer radius growth rate, the rolling time decreases. Thus, the heat loss caused by convection and radiation decreases but the minimum temperature increases. It also can be seen that, with the increase of the ring's outer radius growth rate, the maximum temperature gradually decreases. This is because, according to ((18)) and with the increase of the ring's outer radius growth rate, the feed amount per revolution increases. The ring becomes more easily forged penetrated; and thus the deformation heat decreases.

In Figures 10 and 11, with the increase of the ring's outer radius growth rate, SDP gradually decreases from 1.4 to 0.95; SDT increases from 35.5 to 38.5 at first and then decreases to the minimum value 34.7. This indicates that, with the increase of the ring's outer radius growth rate, the uniformities of PEEQ and temperature distributions increase. When the ring's outer radius growth rate is 6 mm/s, the PEEQ and temperature distributions of the rolled conical ring are the most uniform simultaneously.

### 5.3. The Effects of Driven Roll's Bottom Radius on PEEQ and Temperature Distributions

**Figure 12:**
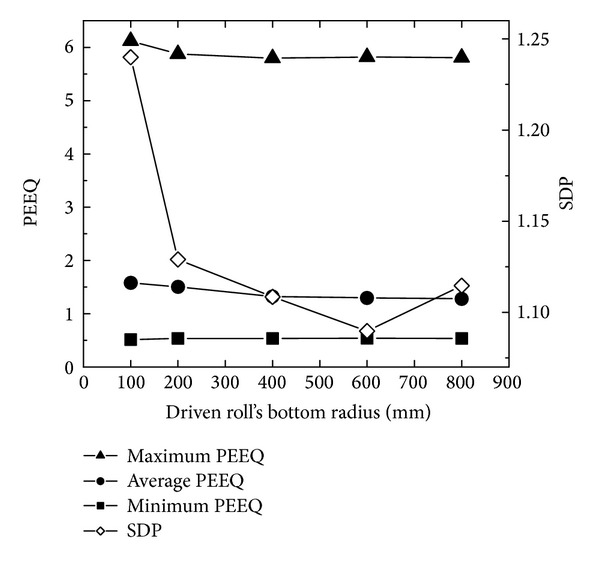
The changing curves of the maximum, minimum, and average PEEQ and SDP with driven roll's bottom radius.

Figure 12 shows the changing curves of the maximum, minimum, and average PEEQ and SDP with driven roll's bottom radius. In Figure 12, with the increase of the driven roll's bottom radius, the minimum PEEQ is basically unchanged; the maximum and average of PEEQ slightly decrease at first and then keep unchanged; SDP decreases rapidly at first and then increases slowly. Based on the conditions of Case 2 and ((18)), when the peripheral speed of the driven roll and ring's outer radius growth rate are unchanged, the feed amount per revolution is invariable. According to ((7)), when the feed amount per revolution is invariable, with the increase of the driven roll's bottom radius from 100 mm to 600 mm, the length of the contact circular arc and the contact areas between the conical ring and the driven roll increase, which results in the ring being more easily forged penetrated. Moreover, with the increase of the driven roll's bottom radius from 600 mm to 800 mm, the contact areas between the conical ring and driven roll further increase, and thus the heat loss between the conical ring and rolls increases, which leads to the increase of material yield strength and the decrease of the forged penetrated ability of the ring.

**Figure 13:**
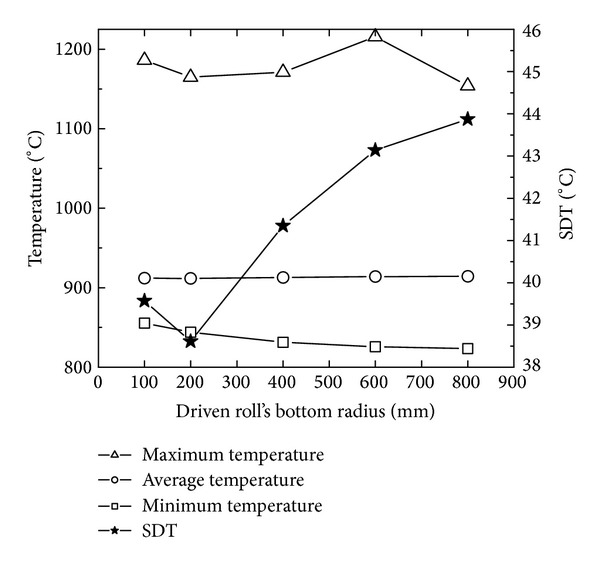
The changing curves of the maximum, minimum, and average temperatures and SDT with driven roll's bottom radius.

Figure 13 shows the changing curves of the maximum, minimum, and average temperatures and SDT with the driven roll's bottom radius. In Figure 13, with the increase of the driven roll's bottom radius, the maximum temperature has a small fluctuation between 1150°C and 1200°C; the minimum temperature gradually decreases; the average temperature is basically invariable; and SDT slightly decreases at first and then gradually increases. The reason may be that, with the increase of the driven roll's bottom radius, the contact area between the conical ring and rolls increases; the heat loss of the rolled ring increases; the minimum temperature decreases; and thus the uniformity of temperature distribution gets worse.

In Figures 12 and 13, with the increase of the driven roll's bottom radius, SDP decreases from 1.24 to the minimum value 1.07 at first and then increases to 1.12; SDT slightly decreases from 39.5 to 38.5 at first and then gradually increases to 44. This indicates that the PEEQ and temperature distributions become uniform at first and then nonuniform. However, the minimum values of SDP and SDT are obtained at different driven roll's bottom radius, respectively. When the driven roll's bottom radius is 200 mm, SDT obtains its minimum value; SDP obtains its relatively minimum value (approximately 1.13); and thus the temperature distribution of rolled conical ring is most homogeneous and the PEEQ distribution of the rolled conical ring is relatively uniform.

### 5.4. The Effects of Mandrel's Bottom Radius on PEEQ and Temperature Distributions

**Figure 14:**
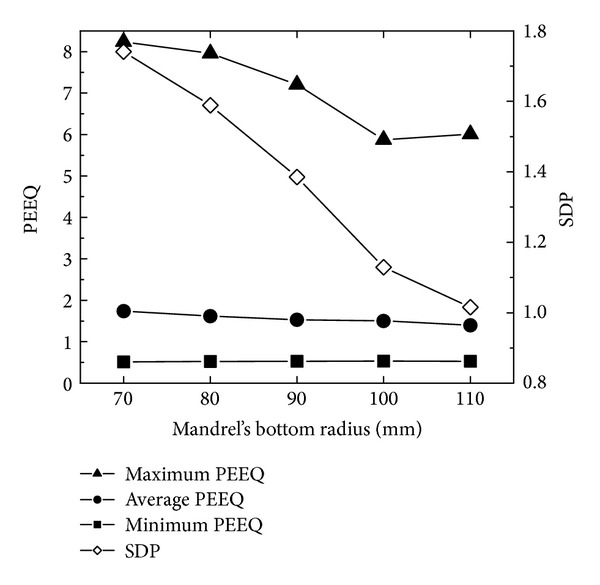
The changing curves of the maximum, minimum, and average PEEQ and SDP with mandrel's bottom radius.

Figure 14 shows the changing curves of the maximum, minimum, and average PEEQ and SDP with mandrel's bottom radius. In Figure 14, with the increase of mandrel's bottom radius, the maximum and average of PEEQ and SDP gradually decrease while the minimum of PEEQ is basically unchanged. This indicates that, with the increase of mandrel's bottom radius, the PEEQ distribution of the rolled ring becomes uniform. The reason is that according to the conditions of Case 3 and ((18)), when both the peripheral speed of the driven roll and the ring's outer radius growth rate are constants, the feed amount per revolution is invariable. According to ((8)), when the feed amount per revolution is invariable, with the increase of mandrel's bottom radius, the length of the contact circular arc and the contact area between the conical ring and mandrel increase, and thus the conical ring becomes more easily forged penetrated.

**Figure 15:**
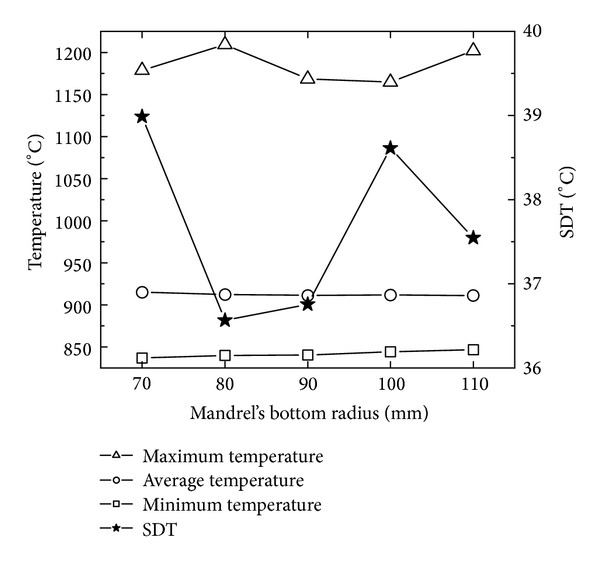
The changing curves of the maximum, minimum, and average temperatures and SDT with mandrel's bottom radius.

Figure 15 shows the changing curves of the maximum, minimum, and average temperatures and SDT with mandrel's bottom radius. In Figure 15, with the increase of mandrel's bottom radius, the minimum and average temperatures are invariable; the maximum temperature has a fluctuation between 1170°C and 1220°C; the SDT fluctuates between 36.5 and 39. This indicates that the effect of mandrel's bottom radius on the temperature distribution of the rolled ring is not obvious.

In Figures 14 and 15, with the increase of mandrel's bottom radius, SDP gradually decreases from 1.75 to the minimum 1.0; and SDT decreases from 39 to the minimum 36.5 at first, then increases, and finally decreases to 37.5. This indicates that, as mandrel's bottom radius increases, the PEEQ distribution becomes more uniform while the uniformity of temperature distribution has a small fluctuation between 36.5 and 39. However, the minimum values of SDP and SDT are obtained at different mandrel's bottom radius, respectively. When mandrel's bottom radius is 110 mm, the PEEQ distribution of rolled conical ring is most uniform and the temperature distribution of the rolled conical ring is relatively uniform.

### 5.5. The Effects of Key Forming Parameters on Average Rolling Force and Rolling Moment

**Figure 16:**
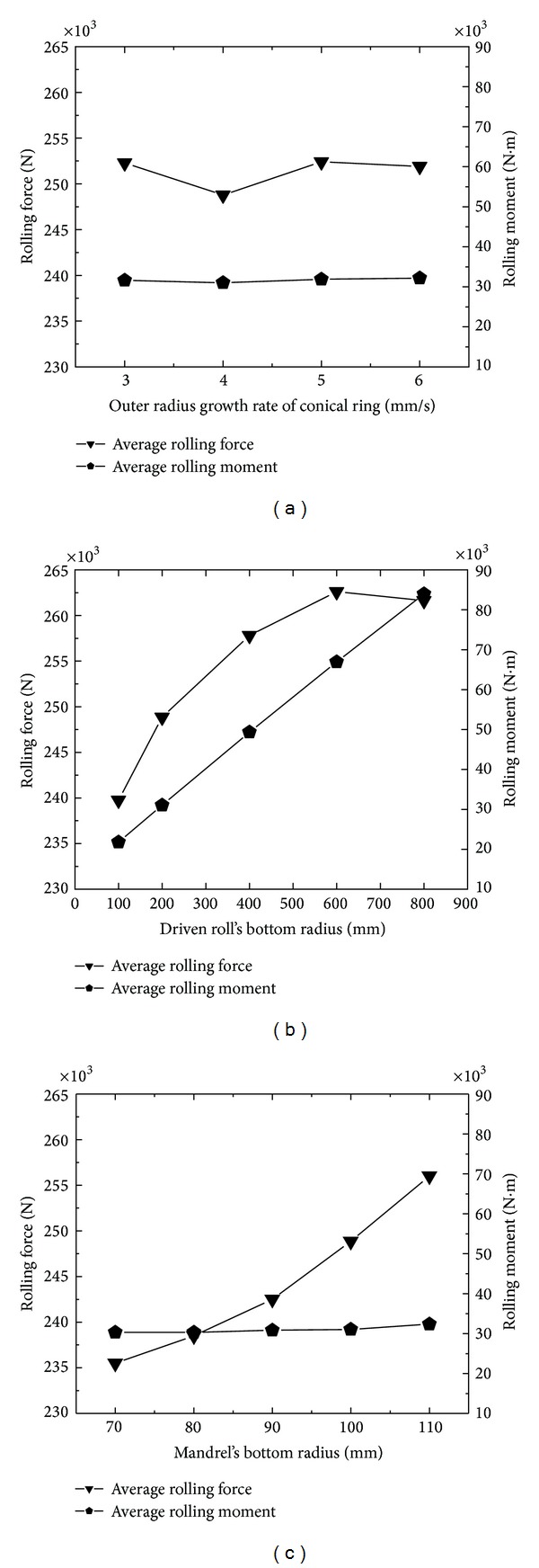
(a) The changing curves of average rolling fore and average rolling moment with ring's outer radius growth rate; (b) the changing curves of average rolling fore and average rolling moment with driven roll's bottom radius; (c) the changing curve of average rolling fore and average rolling moment with mandrel's bottom radius.

Figure 16(a) shows the changing curves of the average rolling force and average rolling moment with the ring's outer radius growth rate. In Figure 16(a), with the increase of the ring's outer radius growth rate, the average rolling force decreases to the minimum 248 kN at first, then increases, and finally keeps unchanged. The reason is that, with the increase of the ring's outer radius growth rate, the feed amount per revolution is basically unchanged, and thus the rolling force changes a little. It also can be seen that, with the increase of the ring's outer radius growth rate, the average rolling moment is basically unchanged, approximately 30 kNm. The reason is that, with the increase of the ring's outer radius growth rate, the force arm is unchanged while the rolling force changes a little, and thus the rolling moment is basically invariable.

Figure 16(b) shows the changing curves of the average rolling force and average rolling moment with the driven roll's bottom radius. In Figure 16(b), with the increase of the driven roll's bottom radius, the average rolling force increases from 240 kN to 263 kN at first and finally slightly decreases to 261 kN. The reason is that, with the increase of the driven roll's bottom radius, the contact area between the conical ring and rolls increases; more metal participates in the plastic deformation; and thus the average rolling force increases. It also can be seen that, with the increase of the driven roll's bottom radius, the average rolling moment basically increases linearly from 20 kNm to 85 kNm. The reason is that, with the increase of the driven roll's bottom radius, both the rolling force and force arm increase; and thus the average rolling moment increases.

Figure 16(c) shows the changing curve of the average rolling force and average rolling moment with mandrel's bottom radius. In Figure 16(c), with the increase of mandrel's bottom radius, the average rolling force gradually increases from 236 kN to 256 kN. The reason is that, with the increase of mandrel's bottom radius, the contact area between the conical ring and rolls increases; more metal participates in the plastic deformation; and thus the average rolling force increases. It also can be seen that, with the increase of mandrel's bottom radius, the rolling moment is basically unchanged, approximately 30 kNm.

To sum up, the radii of the driven roll and mandrel have significant effects on the average rolling force; the driven roll's radius has significant influences on average rolling moment; but the ring's outer radius growth rate slightly affects the average rolling force and average rolling moment.

## 6. Conclusions

This paper established a mathematical model of RCRRCDS process, deduced the plastic penetration condition and biting-in condition, and determined the reasonable value range of the mandrel feed rate in the entire rolling process. The effects of the ring's outer radius growth rate and the radii of the driven roll and mandrel on the PEEQ and temperature distributions, average rolling force, and average rolling moment were investigated. The main conclusions are drawn as follows.

1. The PEEQ at IL and OL of rolled ring are larger than that at ML of the rolled ring. Temperatures at the “obtuse angle zone” of the ring's cross-section are higher than those at “acute angle zone.” The temperature at the central part of the conical ring is more uniform, while the temperatures at the corners of ring's cross-section change significantly.
2. With the increase of the ring's outer radius growth rate at its reasonable value range, the uniformities of PEEQ and temperature distributions increase; the average rolling force fluctuates between 253 kN and 257 kN; and the average rolling moment is unchanged basically. When the conical ring's outer radius growth rate equals 6 mm/s, the PEEQ and temperature distributions of the rolled conical ring are most uniform simultaneously.
3. With the increase of the driven roll's bottom radius, the PEEQ and temperature distributions become more uniform at first and then nonuniform; both the average rolling force and average rolling moment increase gradually. However, the minimum values of SDP and SDT are obtained at different driven roll's bottom radius, respectively. When the driven roll's bottom radius is 200 mm, the temperature distribution of the rolled conical ring is most uniform; the PEEQ distribution of the rolled conical ring is relatively more uniform; and both the average rolling force and average rolling moment are relatively smaller.
4. With the increase of mandrel's bottom radius, the uniformity of PEEQ distribution increases, while the uniformity of temperature distribution changes a little, and the temperature fluctuation range is only 36.5–39°C. With the increase of mandrel's bottom radius, the average rolling force gradually increases, while the average rolling moment is basically unchanged. However, the minimum values of SDP and SDT are obtained at different mandrel's bottom radius, respectively. When mandrel's bottom radius equals 110 mm, the PEEQ distribution of the rolled conical ring is most uniform; the temperature distribution of the rolled conical ring is more uniform; and the corresponding rolling force is 255 kN.
5. The optimal combinations of forming parameters that can obtain more uniform PEEQ and temperature distributions of this kind of middle size titanium alloy conical rings are as follows: the conical ring's outer radius growth rate is 6 mm/s; the driven roll's bottom radius is 200 mm; and mandrel's bottom radius is 110 mm. The results of this study can provide guidance for the selection of ring's outer radius growth rate and rolls sizes.

## Figures and Tables

**Table 1:** Constitutive data for Ti-6Al-4V.

Coefficient	Value
*k*	4.69 × 10^−4^
*m*	0.3
*n*	1
*β*	66.73
*λ*_0_	6.424
*q*	−5.17 × 10^−2^
*α*	6.07
*Q*_defm_/*R*	5.0668 × 10^4^

**Table 2:** Forming parameters in RCRRCDS process.

Parameters	Value
Bottom radius of guide roll (mm)	40
Initial bottom outer radius of ring (mm)	300
Initial bottom inner radius of ring (mm)	150
Initial height of ring (mm)	86.5
Angle between ring and horizontal plane (°)	60
Total feed amount (mm)	70
The peripheral speed of driven roll (mm/s)	1000

**Table 3:** Experimental parameters of RCRRCDS process in [15].

Parameters	Values
Diameters of driven roll's big and small ends (mm)	340.00, 299.60
Diameters of mandrel's big and small ends (mm)	44.52, 5.00
Diameters of guide roll's big and small ends (mm)	100.00, 29.50
Outer and inner diameters of initial conical ring blank's big ends (mm)	100.60, 64.40
Outer and inner diameters of initial conical ring blank's small ends (mm)	60.20, 24.00
Heights of driven roll, mandrel, and guide roll (mm)	80.00
Height of initial conical ring blank (mm)	80.00
Rotational speed of driven roll (r/min)	122
Mandrel feed rate (mm)	2.00

**Table 4:** Experimental and simulation values of rolled ring.

	Outer diameter of small end (mm)	Inner diameter of small end (mm)	Outer diameter of big end (mm)	Inner diameter of big end (mm)	Height (mm)	Taper angle (°)
Experimental values [15]	115	94	167	145	76	71.1
Simulation values	114	93	175	154	76.5	68.3
Numerical error	−0.87%	−1.1%	4.8%	6.2%	0.7%	−3.9%
